# Logistic regression has similar performance to optimised machine learning algorithms in a clinical setting: application to the discrimination between type 1 and type 2 diabetes in young adults

**DOI:** 10.1186/s41512-020-00075-2

**Published:** 2020-06-04

**Authors:** Anita L. Lynam, John M. Dennis, Katharine R. Owen, Richard A. Oram, Angus G. Jones, Beverley M. Shields, Lauric A. Ferrat

**Affiliations:** 1 Institute of Biomedical and Clinical Science, College of Medicine and Health, University of Exeter, Exeter, EX2 5DW UK; 2 Oxford Centre for Diabetes Endocrinology and Metabolism, University of Oxford, Churchill Hospital, Oxford, OX3 7LE UK; 3 Oxford NIHR Biomedical Research Centre, Oxford University Hospitals Foundation Trust, John Radcliffe Hospital, Oxford, UK

**Keywords:** Machine learning, Logistic regression, Model selection

## Abstract

**Background:**

There is much interest in the use of prognostic and diagnostic prediction models in all areas of clinical medicine. The use of machine learning to improve prognostic and diagnostic accuracy in this area has been increasing at the expense of classic statistical models. Previous studies have compared performance between these two approaches but their findings are inconsistent and many have limitations. We aimed to compare the discrimination and calibration of seven models built using logistic regression and optimised machine learning algorithms in a clinical setting, where the number of potential predictors is often limited, and externally validate the models.

**Methods:**

We trained models using logistic regression and six commonly used machine learning algorithms to predict if a patient diagnosed with diabetes has type 1 diabetes (versus type 2 diabetes). We used seven predictor variables (age, BMI, GADA islet-autoantibodies, sex, total cholesterol, HDL cholesterol and triglyceride) using a UK cohort of adult participants (aged 18–50 years) with clinically diagnosed diabetes recruited from primary and secondary care (*n* = 960, 14% with type 1 diabetes). Discrimination performance (ROC AUC), calibration and decision curve analysis of each approach was compared in a separate external validation dataset (*n* = 504, 21% with type 1 diabetes).

**Results:**

Average performance obtained in internal validation was similar in all models (ROC AUC ≥ 0.94). In external validation, there were very modest reductions in discrimination with AUC ROC remaining ≥ 0.93 for all methods. Logistic regression had the numerically highest value in external validation (ROC AUC 0.95). Logistic regression had good performance in terms of calibration and decision curve analysis. Neural network and gradient boosting machine had the best calibration performance. Both logistic regression and support vector machine had good decision curve analysis for clinical useful threshold probabilities.

**Conclusion:**

Logistic regression performed as well as optimised machine algorithms to classify patients with type 1 and type 2 diabetes. This study highlights the utility of comparing traditional regression modelling to machine learning, particularly when using a small number of well understood, strong predictor variables.

## Background

There is much interest in the use of prognostic and diagnostic prediction models in all areas of clinical medicine including cancers [1, 2], cardiovascular disease [3, 4] and diabetes [5, 6]. These models are increasingly being used as web-calculators [7–9] and medical apps for smartphones [10–12], and many have been incorporated into clinical guidelines [13–17].

There are many different approaches that can be used for developing these models. Classic statistical models such as logistic regression are commonly applied but there is increasing interest in the application of machine learning to improve prognostic and diagnostic accuracy in clinical research ([18–21] with many examples of their use [22]. Machine learning (ML) is a data science field dealing with algorithms in which computers (the machines) adapt and learn from experience (data), these algorithms have the ability to process the vast amounts of data such as medical images, biobank and electronic health care records. Supervised learning is the most widely employed category of machine learning. In supervised learning, the machine predicts the value of an outcome (either binary or continuous) trained on a set of predictor variables.

There are many applied studies comparing the performance of classic models to different machine learning algorithms [23–34] but their findings are inconsistent. Many such comparison studies have limitations; not all use non-default parameter settings (hyperparameter tuning) or have validated performance on external data [35]. Discrimination, as measured by area under the receiver operating characteristic curve, is almost always provided but studies have rarely assessed whether risk predictions are reliable (calibration) [35].

We aimed to use a methodological approach to explore and compare the performance of machine learning and a classic statistical modelling approach using an example of a diabetes classification model. Classification of diabetes offers an interesting case study as it is an area where there is considerable misclassification in clinical practice. Type 1 diabetes and type 2 diabetes can be hard to distinguish between, particularly in adults. Correct classification is really important for the patient, particularly in terms of treatment. People with type 1 require insulin injections to prevent life-threatening diabetic ketoacidosis, whereas people with type 2 diabetes can treat their high blood glucose with diet or tablets.

## Methods

We selected a classic model, logistic regression (LR) with linear effects only, and six supervised machine learning algorithms that (1) were appropriate for classification problems and (2) had been used previously in medical applications: gradient boosting machine (GBM), multivariate adaptive regression spline (MARS), neural network (NN), k-nearest neighbours (KNN), random forest (RF) and support vector machine (SVM). We trained models using each algorithm, incorporating hyperparameter tuning, and compared the performance of the optimised models on a separate external validation dataset.

### Study population—training dataset

The Exeter cohort includes 1378 participants, with known diabetes (identified from the clinical record and confirmed by the participant on recruitment) from Exeter, UK [36–39]. Participants with gestational diabetes, known secondary or monogenic diabetes or a known disorder of the exocrine pancreas were excluded. Summaries of the cohorts including recruitment and data collection methods are shown in Supplementary Table 1 and Figure S1 (see Additional file 1).

### Study population—external validation dataset

Five hundred sixty-six participants were identified from the Young Diabetes in Oxford (YDX) study [40]. Participants were recruited in the Thames Valley region, UK, and diagnosed with diabetes up to the age of 50 years. The same eligibility criteria were applied to this cohort.

All participants included in this study (internal and external validation datasets) were of white European origin. Summaries of the cohort including recruitment and data collection methods are shown in Supplementary Table 1 (see Additional file 1).

### Model outcome: type 1 and type 2 diabetes definition

We used a binary outcome of type 1 or type 2 diabetes. Type 1 diabetes was defined as having insulin treatment within ≤ 3 years of diabetes diagnosis and severe insulin deficiency (non-fasting C peptide < 200 pmol/L). Type 2 diabetes was defined as either (1) no insulin requirement for 3 years from diabetes diagnosis or (2) where insulin was started within 3 years of diagnosis, substantial retained endogenous insulin secretion (C-peptide > 600 pmol/L) at ≥ 5 years diabetes duration. Participants not meeting the above criteria or with insufficient information were excluded from analysis, as the type of diabetes and rapid insulin requirement could not be robustly defined (*n* = 342 in the training dataset). These exclusions are unavoidable and in our opinion are unlikely to introduce systemic bias or affect the main question being addressed which is comparative performance of the different modelling approaches. The major reason for exclusion from analysis was short diabetes duration (223 of 342 excluded), and this is because the outcome (based on that the development of severe insulin deficiency is often absent at diagnosis in T1D) cannot be defined in recent onset disease. A tiny number of participants are excluded due to intermediate C-peptide which means outcome cannot be robustly defined (*n* = 37). In 87 participants, a saved serum sample for C-peptide measurement was not available, because serum was not stored in the very early stages of the DARE study. C-peptide was measured in all other participants in these cohorts that required measurement for the outcome.

### Predictor variables

We used seven pre-specified predictor variables, age at diagnosis, BMI, GADA islet-autoantibodies, sex, total cholesterol, HDL cholesterol and triglycerides. Age at diagnosis and sex were self-reported by the participant. Height and weight were measured at study recruitment by a research nurse to calculate BMI. Total cholesterol, HDL cholesterol and triglycerides were extracted from the closest NHS record. Continuous variables were standardised [41]. GADA islet-autoantibodies were dichotomized into negative or positive based on clinically defined cut-offs, in accordance with clinical guidelines [42].

We removed all observations with missing predictor values (complete-case analysis), respectively: 74 for the training cohort (74 HDL cholesterol and 68 triglycerides values missing) and 61 for the external validation cohort (53 sex value missing, 8 total cholesterol missing). We finally removed any observation with clinically impossible values (*z* score > 50): 2 for the training cohort and 1 for the external validation cohort. Nine hundred sixty participants met inclusion criteria and were included in the training dataset, of whom 135 (14%) were classified as having type 1 diabetes. Five hundred four participants (type 1 diabetes, *n* = 105 (21%)) in the YDX cohort met criteria and were included in the external validation dataset. Compared to the participants in Exeter cohort, the participants in the YDX cohort were younger at diagnosis (median 37 years vs 43 years, *p* < 0.001), had a lower BMI (median 31 kg/m2 vs 33 kg/m^2^, *p* < 0.001), had a higher percentage of GADA (20% versus 13%, *p* < 0.001) and a higher prevalence of type 1 diabetes (as defined by our model outcome definition in the ‘Study population—external validation dataset’ section) (21% vs 14%, *p* < 0.001) (Supplementary Table 2 (see Additional file 1) for participant characteristics).

### Model training

All models were trained using the entire training dataset. We evaluated seven classification algorithms: gradient boosting machine (GBM), logistic regression (LR), multivariate adaptive regression spline (MARS), neural network (NN), k-nearest neighbours (KNN), random forest (RF) and support vector machine (SVM). For SVM, we used the radial basis function kernel parameter [41], and for NN, we used the most commonly used single-hidden-layer neural network [41] trained using quasi-Newton back propagation (BFGS) [43] optimisation method. There are no clear guidelines regarding either the choice of algorithms or the advantages and disadvantages of each in specific clinical settings. A brief summary of each algorithm is shown in Table 1.

**Table 1:** Algorithm description and references

Algorithm	Description	References
Logistic regression	A classic statistical algorithm for binary outcomes that use maximum likelihood estimation. It is fully parametric. There are no model hyperparameters to be set. Coefficients are adjusted to allow for dependence between the characteristics. It is useful for inference, estimation, interpretation and prediction.	[41, 44–46]
Random forest	An algorithm that grows a large ensemble of classification trees on bootstrapped samples using a random selection of the predictor variables and performs bagging for class selection; after all the trees have been grown, the predicted class is determined from the average estimated class probability calculated over the ensemble of trees.	[41, 47, 48]
Gradient boosting machine	An ensemble learning technique similar to random forest in the sense they average a large number of decision trees to make prediction. The difference between the two is the application of gradient boosting. In gradient boosting, the decision trees are trained sequentially with the weights of each successive model adjusted based on reducing the errors of the previous model. The predicted class is determined from the average estimated class probability (or majority vote of predicted class) calculated over the ensemble of trees.	[41, 49, 50]
Multivariate adaptive regression spline	MARS and logistic regression share similarities. For the logistic regression model, the logarithm of the odds is fitted with a linear combination of the predictors. For the MARS model, the logarithm of the odds is fitted with splines to cover non-linear and interactions terms. The hinge function (sometimes called rectifier) is used to model the splines.	[51]
Neural network	A method using an adaptive and non-sequential approach to learning that mimics a biological neural network. It is a non-parametric technique where signals travel from the first layer (the input layer), to the last layer (the output layer). Each layer is made of a set of neurons. The output of each neuron is computed by some non-linear function of the sum of its weighted inputs from neurons from the previous layer. The weight increases or decreases the strength of the signal at a connection.	[41, 52–55]
K-nearest neighbours	A model-free method; it is a type of instance-based learning or lazy learning in which there is no training phase, instead the algorithm memorises the training data. Based on the principle that observations located close together in n-dimensional space will have the same outcome, the classification process involves a search the entire dataset for the k training points closest in Euclidean distance (k-neighbours), the probability predicted class is determined based on the average vote of the actual class among these k-neighbours.	[41, 53, 56, 57]
Support vector machine	It is a quadratic optimisation problem involving minimising penalties and maximising margin width, and the two classes are separated by constructing nonlinear decision boundaries (hyperplanes) using a kernel trick that maximises the margin between them. The produced posterior estimates are a rescaled version of the original classifiers scores through a logistic transformation.	[41, 58, 59]

We used a grid search to tune the model parameters (hyperparameter tuning) [60], i.e. optimise the performance of the machine learning algorithm. The hyperparameter metrics applied in the grid searches are shown in Supplementary Table 3 (see Additional file 1). To fit the models over the whole training dataset, we first estimated the hyperparameters with 5-fold cross-validation and fit the models with the estimated algorithms. Internal validation was performed using nested cross-validation. The nested cross-validation consists of an inner loop cross-validation nested in an outer cross-validation. The inner loop is responsible for model selection/hyperparameter tuning (similar to validation set), while the outer loop is for error estimation (test set). For each loop, we used 5 folds. Nested cross-validation is only used to estimate the performance measures, and the final model is fitted on the whole training dataset.

Optimal models were selected using the maximum mean area under the receiver operating characteristic curve (ROC AUC) calculated in the cross-validation. Supplementary Table 3 (see Additional file 1) includes the final model tuning parameters selected for the optimal models in the cross-validation resampling. We computed the 95% CI by assuming that the variation around the mean is normally distributed and computed a standard normalised interval using the different values estimated on each fold computed by the cross-validation.

### Model performance measures

We used ROC AUC [61] as the summary metrics to evaluate model discrimination. The ROC AUC quantifies the probability that the risk scores from a randomly selected pair of individuals with and without this condition are correctly ordered. A value of 1 indicates a perfect test.

We assessed calibration visually using calibration plots, computing calibration performance measure, i.e. calibration slope (the closer to 1 the better) and the calibration in the large (the closer to zeros the better). The slope coefficient beta of the linear predictors reflects the deviations from the ideal slope of 1.

We compared the performance of the model to support decision-making with decision curve analysis [62]. In decision curve analysis, a clinical judgement of the relative value of benefits (treating a true-positive case) and harms (treating a false-positive case) associated with prediction models is made for different threshold probability [63]. The net benefit is computed by subtracting the proportion of all patients who are false-positive from the proportion who are true-positive, weighting by the relative harm of a false-positive and a false-negative result.

### External testing

For each optimal model developed in the training dataset, external performance was evaluated in the YDX study cohort and compared to the internal (cross-validation resampling) performance. Calibration was investigated using calibration curves. We also checked for Pearson’s correlation in the predictions from each model.

### Software

All analyses were performed using R software (version 3.5.2). Model training was performed using the Caret R package [64–68].

### Code

In the supplementary material, we share the code to allow reproduction of similar comparisons of machine learning algorithms with any number of predictor variables (see Additional file 2).

## Results

The average (mean) performance ROC AUC for the optimal models obtained in the resampling was high in all models (ROC AUC ≥ 0.93) (Table 2) with small difference in performance between models.

**Table 2:** ROC AUC [95% CI] performance comparison of the seven models applied to the internal and external validation datasets. Internal validation was estimated with 5-fold-nested cross-validation while external validation was performed on the YDX dataset

Model	Internal validation	External validation
Gradient boosting machine	0.96 [0.94, 0.98]	0.93 [0.90, 0.96]
K-nearest neighbours	0.93 [0.90, 0.97]	0.92 [0.89, 0.95]
Logistic regression	0.96 [0.93, 0.98]	0.95 [0.92, 0.97]
MARS	0.96 [0.90, 0.99]	0.94 [0.92, 0.97]
Neural network	0.96 [0.93, 0.99]	0.94 [0.92, 0.97]
Random forest	0.95 [0.92, 0.98]	0.94 [0.91, 0.96]
Support vector machine	0.96 [0.93, 0.98]	0.94 [0.92, 0.97]

There was a decrease in the ROC AUC of all models when they were applied to the external validation dataset (Table 2), but all still showed high levels of performance (ROC AUC ≥ 0.92, Figure S3) for all model. Model predictions were highly correlated across models (Figure S2 (see Additional file 1)). ROC AUC performance was similar when fitting the model with or without resampling.

**Fig. 1:**
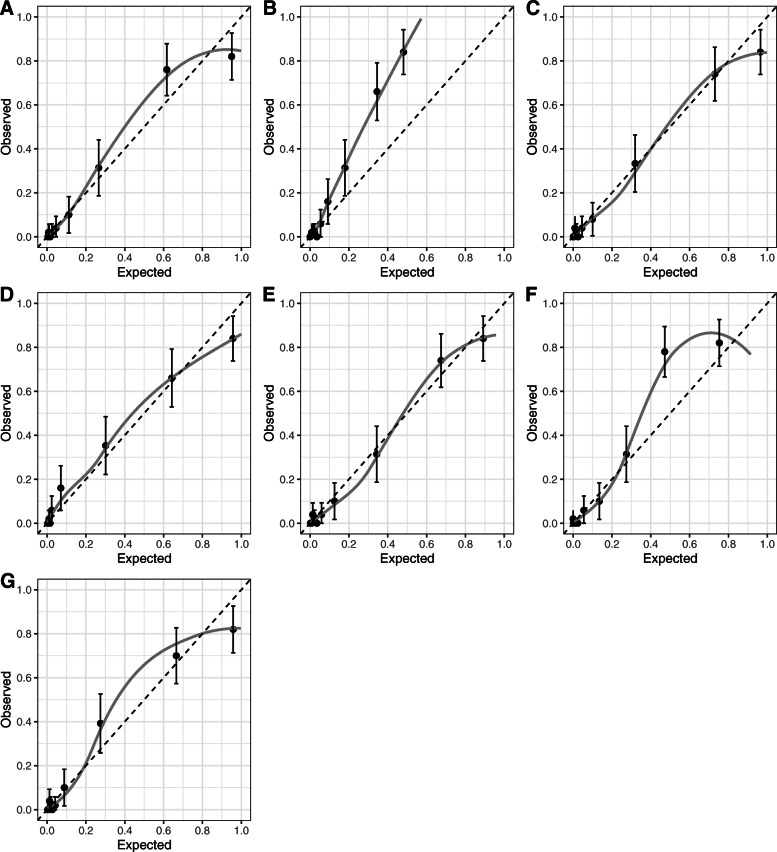
Calibration plots with 95% confidence interval obtained using external validation dataset for prediction models. **a** Gradient boosting machine. **b** K-nearest neighbours. **c** Logistic regression. **d** MARS. **e** Neural network. **f** Random forest. **g** Support vector machine. Legend: Dashed line = reference line, solid black line = linear model

In the calibration tests performed on the external validation dataset, GBM and NN shows very good calibration performance with a calibration in the large close to 0 and a calibration slope close to 1. Logistic regression and support vector machine have a satisfactory calibration results but the likelihood to predict type 1 diabetes is on average slightly underestimated. All other models have unsatisfactory calibration performance (Fig. 1 and Table 3 (calibration in the large values < 0 indicate over-estimating risk)) and there was evidence of visual miscalibration in these models (often due to an underestimation of type 1).

**Table 3:** Calibration test results on external validation dataset. Calibration-in-the-large indicates whether predicted probabilities are, on average, too high (value below 0) or too low (value above 0). Conversely, the calibration slope quantifies whether predicted risks are, on average, too extreme (value below 1) or too invariant (value above 1)

Model	Calibration slope (***b***_***L***_)	Calibration-in-the-large (***a***|***b***_***L***_ = 1)
Gradient boosting machine	0.979	− 0.005
K-nearest neighbours	1.495	0.046
Logistic regression	0.903	− 0.039
MARS	0.799	0.081
Neural network	0.995	− 0.031
Random forest	1.412	0.065
Support vector machine	0.914	− 0.028

Figure S3 highlights that the most performing machine learning algorithms give similar predictions. In this figure, the prediction for each algorithm is plotted for each observation in XYD. SVM, NN and LR predictions are strongly correlated (LR-NN, 0.992; LR-SVM, 0.99; NN-SVM, 0.983). For all models, the majority of predicted probability is below 0.3 (as expected, 79% of people do not have type 1 diabetes). Excepted for the KNN model, few predictions lie between 0.3 and 0.7.

**Fig. 2:**
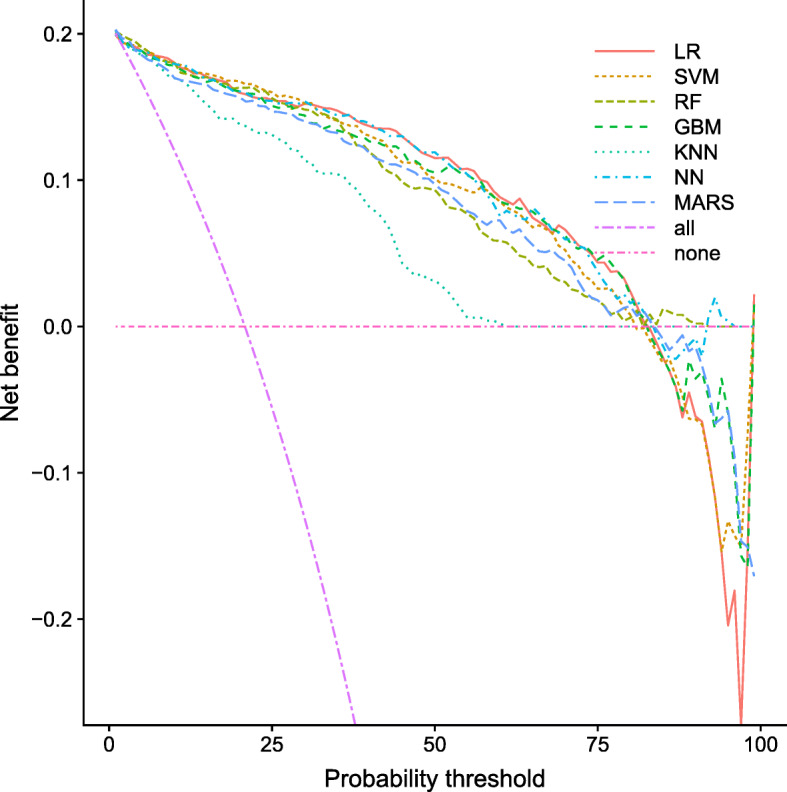
Decision curve analysis obtained using external validation dataset for prediction models. The graph gives the expected net benefit per patient relative to treat all patients as type 2 diabetes. The unit is the benefit associated with one patient with type 1 diabetes receiving the correct treatment. ‘all’: assume all patients have type 1 diabetes. ‘none’: assume no patients have type 1 diabetes

Figure 2 is the decision curve analysis where the net benefit is plotted against the threshold probability. The LR model is superior or similar to the other models across a wide range of threshold probabilities but becomes worse than the other models for higher threshold probabilities. In practice, it is likely clinicians would be cautious and treat patients with insulin at much lower probabilities threshold, as not receiving correct treatment for a type 1 diabetes can be life-threatening while giving insulin to a patient with type 2 diabetes is inconvenient and expensive but not life-threatening.

The poor performance at high threshold probabilities is due to the fact that the LR model, like the SVM model, tends to overestimate the risk of having type 1 diabetes for people with the highest risk (risk above 85%), see Fig. 1.

## Discussion

### Summary of main findings

We found similar performance when applying logistic regression and six optimised machine learning algorithms to classify type 1 and type 2 diabetes, in both internal and external validation datasets. Discrimination was high for all models, while logistic regression showed the numerically highest discrimination in external validation differences in discrimination were small. Neural network and gradient boosting machine had the best calibration performance, with logistic regression and support vector machine also showing satisfactory calibration.

### Strengths and limitations

Strengths of our study include the use of a systematic approach to model comparison dealing with limitations from previous studies [35, 70] including (1) use of different datasets to train and test models, (2) optimisation of tuning parameters [24, 30], (3) calibration [18] and (4) decision curve analysis. We have used the same dataset to train all our models; since model performance will differ between settings, the use of the same dataset is crucial for valid model comparisons. The choice of tuning parameters will affect the performance of the model [60], and we have optimised our models by applying hyperparameter tuning using a recognised grid search approach. We have increased the validity of our results by using an external validation dataset.

We have compared several machine learning algorithms that have been selected for their suitability to our setting. The use of only seven predictor variables means that we have a very low risk of over-fitting; for machine learning algorithms, it has been suggested that over ten times as many events per variable is required to achieve stable results compared to traditional statistical modelling [69]. The use of only seven predictors may also be considered as a limitation of our study since these machine learning algorithms are designed to deal with larger datasets and more variables. However, working with a few meaningful predictors is common in clinical settings. Knowing the performance of machine learning models using low numbers of predictors is important. It is possible that with more variables or more observations, machine learning approaches may prove more discriminative. However, we have achieved excellent performance using just these seven predictors. Another limitation of our study is that we judge the model only on its performance. In real practice, we would want to consider ease of implementation and interpretation when selecting the ‘best’ model.

LR, SVM and NN are the models with the highest ROC AUC. If accuracy of estimated probability were an importance factor, NN, LR, GBM and SVM would be best approaches. Overall, the notion of best model is context-dependent, but in this study, the models perform similarly. In terms of clinical utility, LR and SVM appeared to perform slightly better than other models.

The observed decrease in ROC AUC when assessed in the external validation data highlights the importance of external validation to test the transportability of models. Indeed, all of the algorithms slightly underperformed in the external validation set. The model fit on the training data set might be over-fitted and their performance could be overestimated despite a rigorous internal validation (see the difference between internal and external performance in Table 2). However, the most likely reason is that the YDX population has a smaller range in age and BMI, and GADA is less discriminative in YDX compared to the Exeter cohort. This may diminish performance and does not necessarily mean over-fitting.

The performance of LR on both internal and external validation datasets shows that classic algorithms can perform as well as more advanced algorithms even when disadvantaged by assuming linearity in the predictors. LR models are relatively easy to use and understand compared to machine learning algorithms where usage is limited by the difficultly of interpreting the model, often referred to as a ‘black boxes’. LR models also have a strong theoretical background which leads to the possibility of using well-defined statistical tests to explore the statistical significance of the variables. There is an increasing number of studies demonstrating that LR can perform as well if not better, in a large number of settings [35]. However, we could not find a study that compared machine learning algorithms with optimised hyperparameters versus LR on an external dataset as we have done in this study which shows again that LR performs as well as more complex approaches.

While real-world data medical applications are likely to be unbalanced, the use of sampling methods such as Synthetic Minority Over-Sampling Technique (SMOTE) might improve model prediction performance [70]. We compared the use of SMOTE to the classic approach without resampling. Nevertheless, we only present the results without SMOTE as similar ROC AUC, but better result calibration and decision curve analysis performance were achieved without it.

We have shown through this study that machine learning performs similarly for this prediction problem; however, some differences subsist. As previously described [71], each database is unique and there is no ‘free lunch’, i.e. if an algorithm performs well on a certain class of problems, then it necessarily pays for that with degraded performance on the set of other problems [35, 72]. It is thus important to test different algorithms benchmarked against logistic regression to identify if one algorithm outperforms the other; if performance is similar, then the simplest and most interpretable model can be used.

## Conclusion

In a diabetes classification setting with three strongly predictive variables, a classic logistic regression algorithm performed as well as more advanced machine algorithms. This study highlights the utility of comparing traditional regression modelling to machine learning, particularly when using a small number of well understood, strong predictor variables. Furthermore, this article highlights once again the need to perform external validation when selecting models as we demonstrate that all algorithms can underperform on external data.

## Supplementary information

**Additional file 1: Figure S1.** Flow diagram of participants through the model development stages. T1D: type 1 diabetes, T2D: type 2 diabetes. **Figure S2.** ROC AUC plots obtained using external validation dataset for seven prediction models. Legend: Solid lines: black = Support Vector Machine, dark grey = Logistic Regression, light grey = Random Forest. Dotted lines: black = Neural Network, dark grey = K-Nearest Neighbours, light grey = Gradient Boosting Machine. **Figure S3.** Correlation coefficient matrix and scatter plot of model predictions obtained from external test validation data.

**Additional file 2.** R script.

## Data Availability

The data that support the findings of this study are available from University of Exeter Medical School/Oxford University but restrictions apply to the availability of these data, which were used under license for the current study, and so are not publicly available. Data are however available from the authors upon reasonable request and with permission of University of Exeter Medical School/Oxford University. R code is made available in supplementary file (see Additional file 2).

## Abbreviations

GADA: Glutamic acid decarboxylase antibodies
YDX: Young Diabetes in Oxford
LR: Logistic regression
SVM: Support vector machine
GBM: Gradient boosting machine
NN: Neural network
KNN: K-nearest neighbours
RF: Random forest
SMOTE: Synthetic Minority Over-Sampling Technique
ROC AUC: Area under the receiver operating characteristic curve
