# Supplementary material for: Logistic regression has similar performance to optimised machine learning algorithms in a clinical setting: application to the discrimination between type 1 and type 2 diabetes in young adults
Source: Diagn Progn Res. 2020 Jun 4;4:6. doi: 10.1186/s41512-020-00075-2 (PMC7318367; doi:10.1186/s41512-020-00075-2)
Supplement: Supplementary file 1 — Additional file 1: Figure S1. Flow diagram of participants through the model development stages. T1D: type 1 diabetes, T2D: type 2 diabetes. Figure S2. ROC AUC plots obtained using external validation dataset for seven prediction models. Legend: Solid lines: black = Support Vector Machine, dark grey = Logistic Regression, light grey = Random Forest. Dotted lines: black = Neural Network, dark grey = K-Nearest Neighbours, light grey = Gradient Boosting Machine. Figure S3. Correlation coefficient matrix and scatter plot of model predictions obtained from external test validation data. [file 41512_2020_75_MOESM1_ESM.zip › figS1R3.pdf]

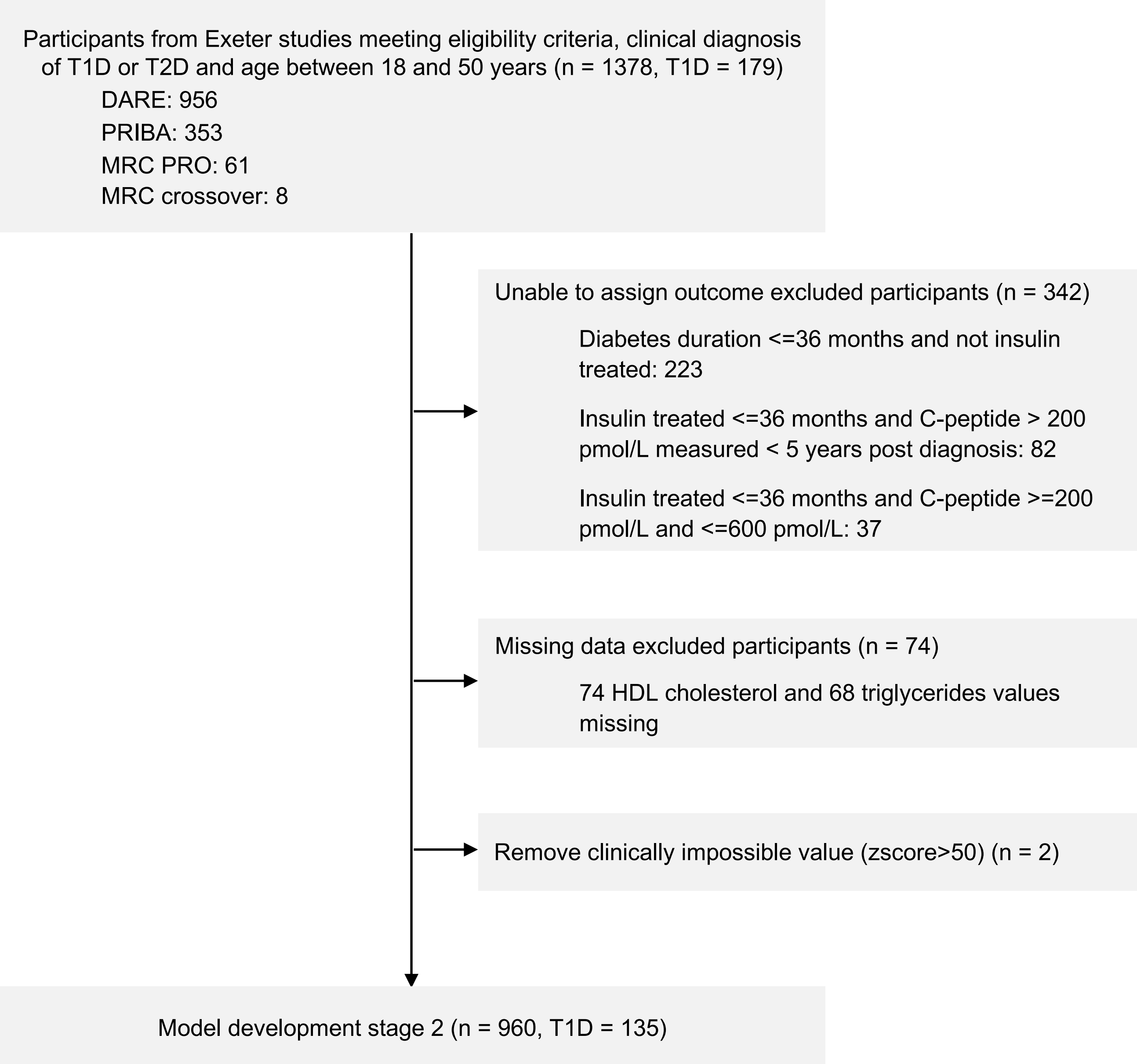
Participants from Exeter studies meeting eligibility criteria, clinical diagnosis of T1D or T2D and age between 18 and 50 years (n = 1378, T1D = 179)
DARE: 956
PRIBA: 353
MRC PRO: 61
MRC crossover: 8
Unable to assign outcome excluded participants (n = 342)
Diabetes duration <=36 months and not insulin treated: 223
Insulin treated <=36 months and C-peptide > 200 pmol/L measured < 5 years post diagnosis: 82
Insulin treated <=36 months and C-peptide >=200 pmol/L and <=600 pmol/L: 37
Missing data excluded participants (n = 74)
74 HDL cholesterol and 68 triglycerides values missing
Remove clinically impossible value (zscore>50) (n = 2)
Model development stage 2 (n = 960, T1D = 135)
