# Supplementary material for: Logistic regression has similar performance to optimised machine learning algorithms in a clinical setting: application to the discrimination between type 1 and type 2 diabetes in young adults
Source: Diagn Progn Res. 2020 Jun 4;4:6. doi: 10.1186/s41512-020-00075-2 (PMC7318367; doi:10.1186/s41512-020-00075-2)
Supplement: Supplementary file 1 — Additional file 1: Figure S1. Flow diagram of participants through the model development stages. T1D: type 1 diabetes, T2D: type 2 diabetes. Figure S2. ROC AUC plots obtained using external validation dataset for seven prediction models. Legend: Solid lines: black = Support Vector Machine, dark grey = Logistic Regression, light grey = Random Forest. Dotted lines: black = Neural Network, dark grey = K-Nearest Neighbours, light grey = Gradient Boosting Machine. Figure S3. Correlation coefficient matrix and scatter plot of model predictions obtained from external test validation data. [file 41512_2020_75_MOESM1_ESM.zip › SupplementaryMaterialcorrected2R3.docx]

|  | **DARE** | **PRIBA** | **MRC Pro/RetroMaster** | **MRC crossover** | **XYD** |
| --- | --- | --- | --- | --- | --- |
| Included participants | 956 | 353 | 61 | 8 | 566 |
| Data collection period | 2007 to 2017 | 2011 to 2013 | 2013 to 2015 | 2013 to 2015 |  |
| Study design | Cross-sectional | Longitudinal | Cross-sectional | Interventional Crossover | Cross-sectional |
| Setting | Primary and secondary care in eight diabetes research regions, England and retinal screening clinics. | Primary and secondary care in South West England | Primary and secondary care sites South West England, Tayside, Oxford, Glasgow, KCL and Newcastle, U.K. | Exeter and Tayside,U.K. | Primary and secondary care in 12 GP surgeries and a search of the hospital clinic database in Thames Valley region |
| Inclusion criteria | Clinical diagnosis of diabetes (any type). | Clinical diagnosis of type 2 diabetes. Clinician determined requirement for DPP-IV inhibitor or GLP-1 analogue (HbA1C >7.5%) | Clinical diagnosis of type 2 diabetes non-insulin treated within 6 months of diagnosis. Age 18-90 inclusive. | Clinical diagnosis of type 2 diabetes, currently treated with sulphonylurea tablets and no change in treatment in previous 3 months, Last HbA1c (within previous 12 months) ≥42 and ≤75 mmol/mol (6-9%).  Age 19-79 inclusive. | Clinical diagnosis of diabetes (any type). |
| Data collection | Clinical measurements and blood sample collected at visit. Ongoing biochemical data collected from pathology laboratories. | Clinical measurements and blood taken at initial visit. Follow up clinical measurements and blood collected at three and six months. | Clinical measures and fasting blood sample taken at visit. | MMT at baseline & MMT on each study drug visits. Three fasting blood collected at crossovers. | Clinical measurements and blood sample collected at visit. Ongoing biochemical data collected from pathology laboratories. |

Supplementary Table 1: Cohort recruitment and data collection methods summary. The training dataset include data from DARE, PRIBA, MRC pro and MRC crossover. The validation dataset includes data from XYD.

|  | **Exeter** | **YDX** |
| --- | --- | --- |
|  | N=960 | N=504 |
| **Type 1 diabetes** | 135 (14.1%) | 105 (20.8%) |
| **Age at Diagnosis** | 43.0 [36.0;48.0] | 37.0 [30.0;41.0] |
| **BMI, Median** | 33.1 [27.8;38.5] | 31.2 [27.4;36.4] |
| **GADPositive975, N (%):** | 123 (12.8%) | 99 (19.6%) |
| **HDL cholesterol** | 1.29 [1.09;1.30] | 1.20 [0.92;1.44] |
| **Male, N (%):** | 567 (59.1%) | 312 (61.9%) |
| **Total Cholesterol** | 4.10 [3.77;4.70] | 4.30 [3.70;5.00] |
| **Triglycerides** | 1.57 [1.57;1.57] | 1.40 [0.96;2.10] |

Supplementary Table 2: Characteristics of the Exeter, U.K. study participants included in the model training and Young Diabetes in Oxford participants included in the model external testing. Median (IQR) or %.

| **Model** | **function in the package caret** | **Grid Search parameter values** | **Final values used for the optimal model** | **Description of hyperparameters** |
| --- | --- | --- | --- | --- |
| Gradient Boosting machine | gbm | n.trees = (20,50,100, 500,2000) interaction.depth = (1, 3, 7, 10)  shrinkage = (from 0.01 to 0.1 by 0.01) n.minobsinnode = (5,10,20,50,200) | n.trees = 50, interaction.depth = 3  shrinkage = 0.07 n.minobsinnode = 5 | n.trees = number of trees  interaction.depth = ﻿ ﻿the highest level of variable interactions allowed  shrinkage = ﻿ learning rate  n.minobsinnode = ﻿minimum number of observations in the terminal nodes of the trees |
| K-Nearest Neighbours | knn | k = (from 1 to 200 by 1) | k = 122 | k = number of neighbours |
| Logistic Regression | glm | N/A | N/A | N/A |
| MARS | earth | degree = (1,2,3)  nprune = (from 1 to 100 by 1) | degree = 1  nprune = 12 | degree = Maximum degree of interaction.  nprune = Maximum number of terms |
| Neural Network | nnet | size = (from 2 to 10 by 1)  decay = (1,0.5, 0.1, 0.01, 0.001, 0.0001, 0.00001, 0.000001, 0.0000001) | size = 2 decay = 0.5 | size = ﻿number of units in the hidden layer  decay = learning rate of back-propagation |
| Random Forest | rf | mtry = (from 1 to number of variables) | mtry = 1 | mtry = Number of variables randomly sampled as candidates at each split |
| Support Vector Machine (with Radial Basis Function Kernel) | svmRadial | sigma = (0.0001,0.001,0.01, 0.1, 1, 10, 100) C = (from 0.1 to 2 by 0.05) | sigma = 0.01 C = 1.9 | Sigma = inverse kernel width  C = the regularization term in the Lagrange formulation |

Supplementary Table 3: Model training details including the R training method used and grid search parameters applied in hyperparameter tuning, and model parameters for the optimal model selected using largest ROC AUC value. There are no model parameters for logistic regression. Hyperparameter tuning was not used for Random Forest due to the low number of predictor variables. Descriptions for search parameters are available in reference. Seed choice was set to 7 in model training.
