# Supplementary figures and images for: Logistic regression has similar performance to optimised machine learning algorithms in a clinical setting: application to the discrimination between type 1 and type 2 diabetes in young adults
Source: Diagn Progn Res. 2020 Jun 4;4:6. doi: 10.1186/s41512-020-00075-2 (PMC7318367; doi:10.1186/s41512-020-00075-2)

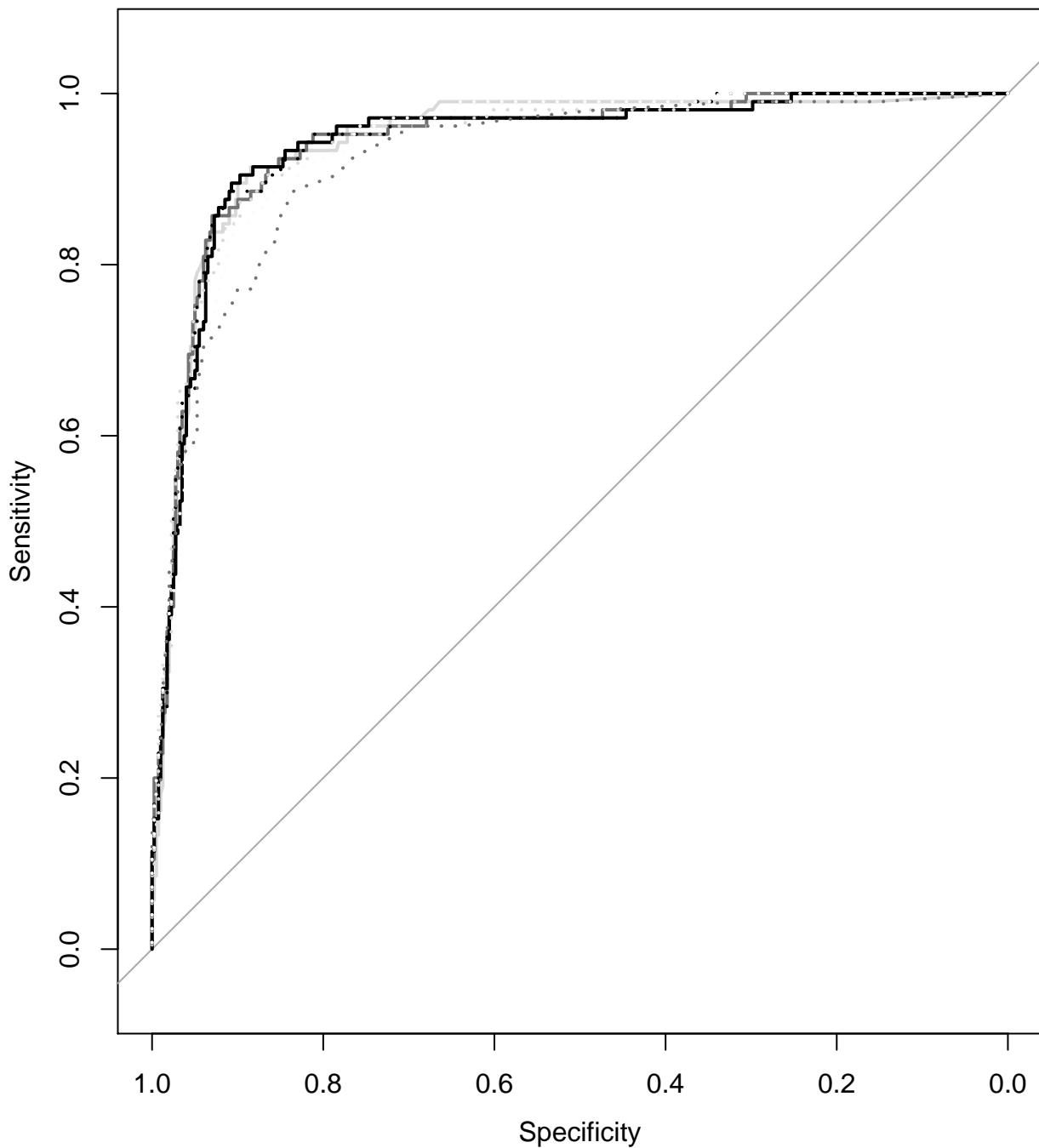

Supplement: Supplementary file 1 — Additional file 1: Figure S1. Flow diagram of participants through the model development stages. T1D: type 1 diabetes, T2D: type 2 diabetes. Figure S2. ROC AUC plots obtained using external validation dataset for seven prediction models. Legend: Solid lines: black = Support Vector Machine, dark grey = Logistic Regression, light grey = Random Forest. Dotted lines: black = Neural Network, dark grey = K-Nearest Neighbours, light grey = Gradient Boosting Machine. Figure S3. Correlation coefficient matrix and scatter plot of model predictions obtained from external test validation data. [file 41512_2020_75_MOESM1_ESM.zip › figS2R3.pdf]

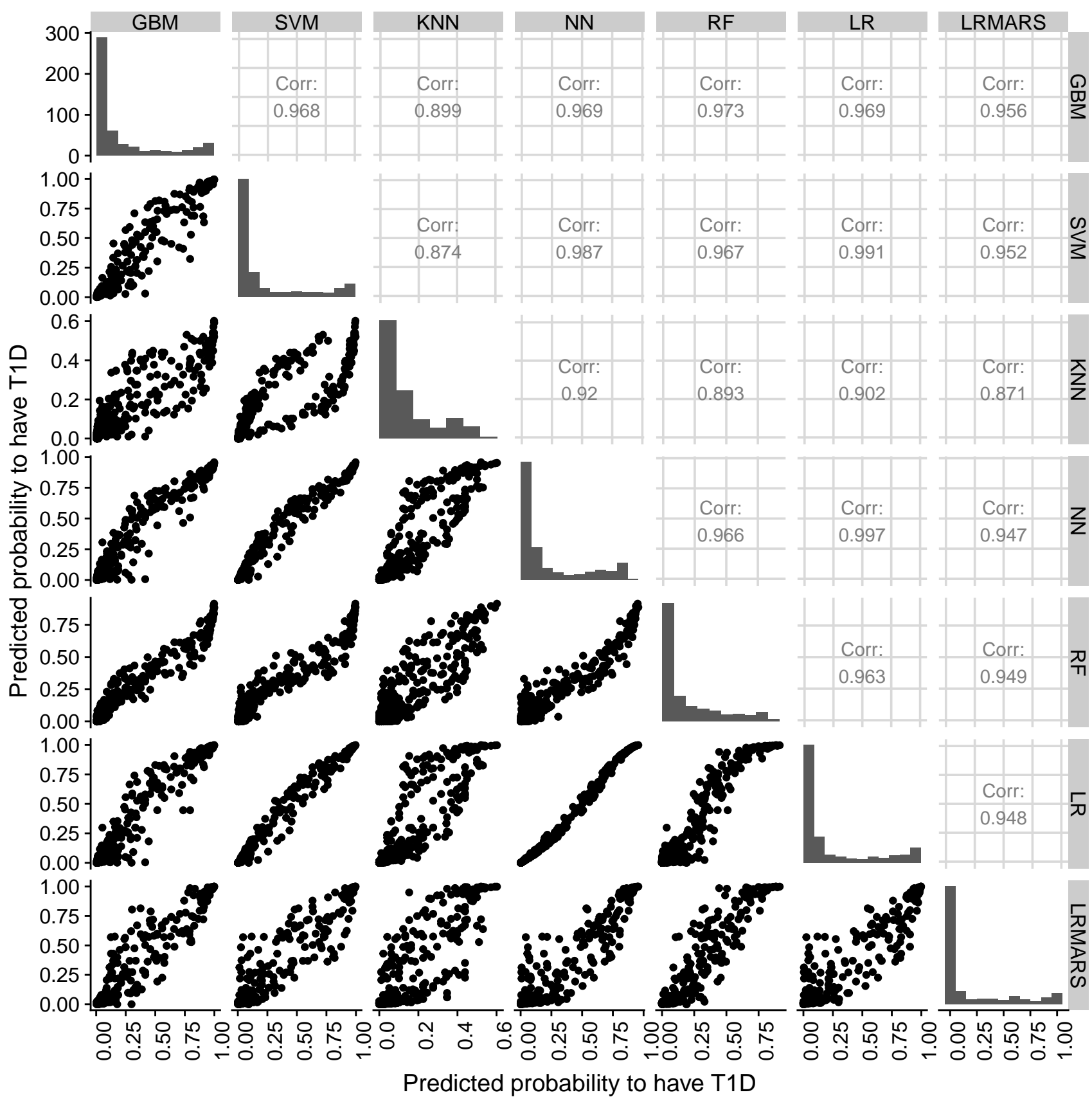

Supplement: Supplementary file 1 — Additional file 1: Figure S1. Flow diagram of participants through the model development stages. T1D: type 1 diabetes, T2D: type 2 diabetes. Figure S2. ROC AUC plots obtained using external validation dataset for seven prediction models. Legend: Solid lines: black = Support Vector Machine, dark grey = Logistic Regression, light grey = Random Forest. Dotted lines: black = Neural Network, dark grey = K-Nearest Neighbours, light grey = Gradient Boosting Machine. Figure S3. Correlation coefficient matrix and scatter plot of model predictions obtained from external test validation data. [file 41512_2020_75_MOESM1_ESM.zip › figs3R3.pdf]
